# CAG Repeat Expansions Increase N^1^-Methyladenine to Alter TDP-43 Phase Separation: Lights Up Therapeutic Intervention for Neurodegeneration

**DOI:** 10.14336/AD.2024.0110

**Published:** 2024-01-10

**Authors:** Lin Yuan, Li-Hong Mao, Jia-Yi Li

**Affiliations:** ^1^Laboratory of Research in Parkinson’s Disease and Related Disorders, Health Sciences Institute, China Medical University, Shenyang, China.; ^2^Neural Plasticity and Repair Unit, Wallenberg Neuroscience Center, Department of Experimental Medical Science, Lund University, BMC 221 84 Lund, Sweden.

**Keywords:** m^1^A, CAG repeat expansion, TDP-43, gel-like aggregates

## Abstract

N^1^-methyladenine (m^1^A), a modification of transcripts, regulates mRNA structure and translation efficiency. In a recent issue of *Nature*, Sun *et al*. reported that m^1^A in CAG repeat RNA contributes to CAG repeat expansion-induced neurodegeneration in *Caenorhabditis elegans* and *Drosophila* through enhancing the ability of endogenous TDP-43 to partition into stress granules mediated by m^1^A. The study is especially important for revealing the pathological function of m^1^A in RNA and the pathological mechanisms of CAG repeat expansion-related neurodegenerative diseases.

Enlightened by the Chinese Creation Myth that Pangu separates the chaotic world into heaven and earth, deciphering the regulatory mechanisms underlying liquid-liquid phase separation (LLPS) is feasible to rehabilitate cellular homeostasis and prevent neurodegeneration. Expansion of microsatellites beyond the threshold can trigger the initiation of over two dozen neurological diseases [1]. RNA molecules with microsatellite repeat expansions can undergo LLPS into gel-like condensate [2]. Searching for the connection between RNA modifications and disease-associated microsatellite repeat expansions will help with the step-by-step characterization of neurodegenerative diseases (NDDs) and improve therapeutic intervention.

In the latest article published in *Nature*, Sun *et al.* reported that repeated expansions of CAG causes higher amounts of N^1^-methyladenine (m^1^A), which binds to TDP-43 and then promotes TDP-43 cytoplasmic mislocalization and gel-like aggregate formation [3]. These findings reveal a novel pathogenic function of m^1^A in RNA as well as a new paradigm of the mechanism for CAG repeat expansion-related NDDs.

CAG abnormal repeat expansions have been documented in multiple NDDs, including Huntington’s disease (HD) and spinocerebellar ataxia (SCA) [4]. It has been reported that replication protein A (RPA1, RPA2, and RPA3) impedes CAG expansions and enhances slipped-CAG repair, but alternative-RPA prevent slipped-CAG repair and increases CAG expansions [5]. However, there are no effective therapeutic treatments for CAG repeat expansions related to NDDs because the pathological mechanisms are complex, and only symptomatic treatments have been used for NDDs till now. Excitingly, several studies have been performed to alleviate pathologies of HD, such as the silencing of Msh3 that blocks somatic repeat expansions in HD mouse models [6]. Although DNA repair pathways are potential therapies for CAG-expansion-related NDDs, other treatment strategies directly targeting RNA or RNA post-transcriptional modifications may be strong-fidelity strategies.

RNA methylation is a post-transcriptional level of regulation, previous studies have focused on the contribution of N^6^-methyladenine (m^6^A) to pathological protein aggregation in NDDs [7]. In addition, there is an emerging appreciation that m^1^A also plays a key role in the relevant studies [8]. m^1^A is installed onto tRNA by the methyltransferases, including TRMT10C, TRMT6-TRMT61A, and TRMT61B. m^1^A on tRNA can be removed by ALKBH1 and ALKBH3 [9]. Given that m^1^A has a wide-range role in the regulation of gene expression, it is required for physiological and pathophysiological processes.

In the recent issue of *Nature*, Sun *et al*. identified the new mechanisms regulating the TDP-43 phase transition mediated by m^1^A. A progressive increase in the frequency of m^1^A with CAG repeat lengths contributes to neurodegeneration. The scientific question is whether m^1^A aggravates neurodegeneration. TRMT61A and ALKBH3 are identified as the natural writers and erasers of m^1^A via knockdown by short hairpin RNA (shRNA) and ectopic expression separately *in vitro*. The decreased level of m^1^A is also identified by the genetic depletion of WO2A11.1, which is the nematode orthologue of the human TRMT61A gene, and neuronal expression of ALKBH3-WT *in vivo*. They showed that neurodegeneration and neurotoxicity could be decreased by decreasing the level of m^1^A.

**Figure 1.**
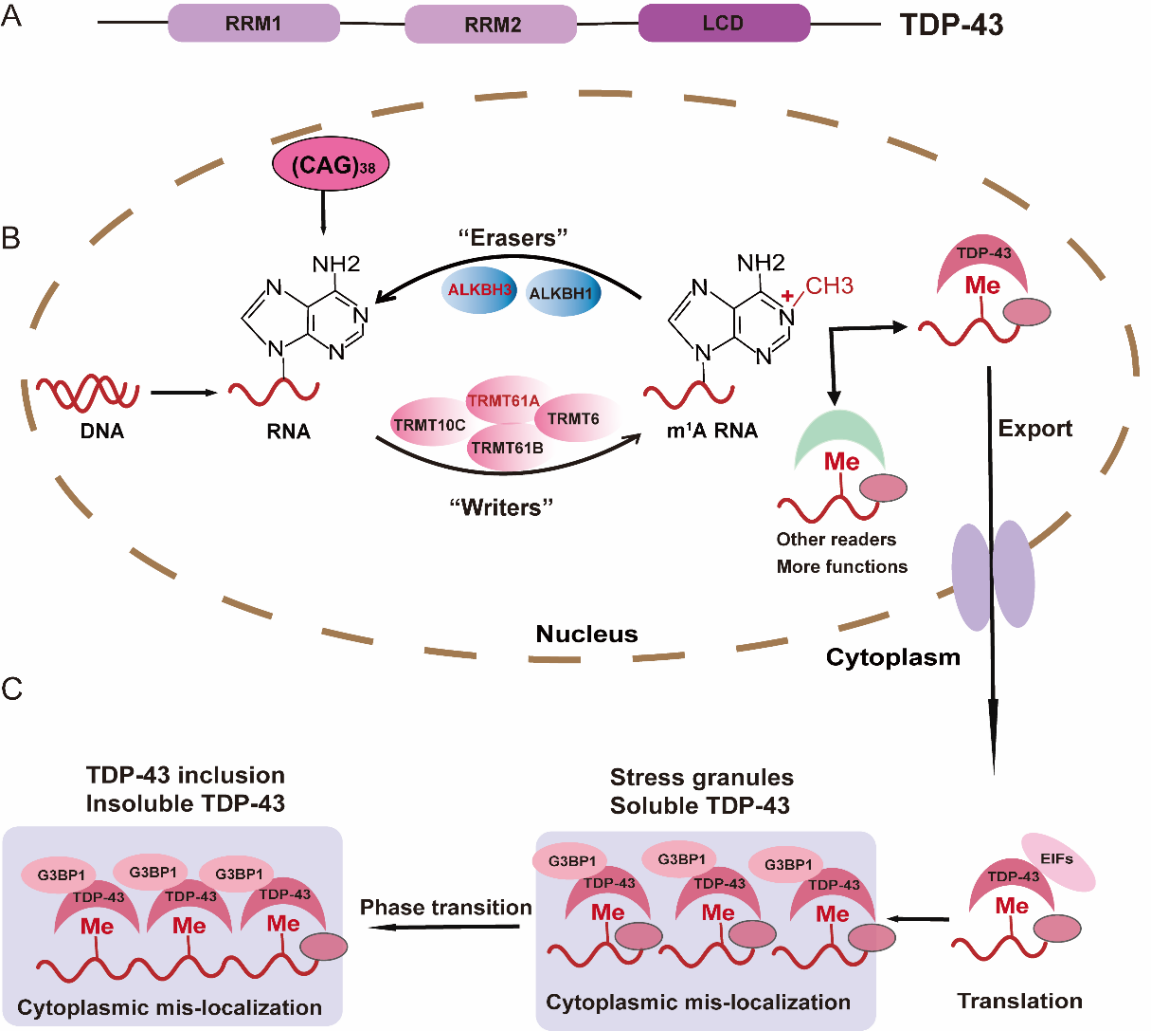
**m^1^A-RNA alters the phase separation of TDP-43**. (**A**) TDP-43 contains two RNA-recognition motifs (RRMs) and a C-terminal low-complexity domain (LCD). CAG repeat RNA, mainly recognized by RRM, then promotes the liquid-to-gel transition of TDP-43 via LCD. (**B**) CAG repeat RNA-carrying synthetic m^1^A causes endogenous TDP-43 to undergo cytoplasmic redistribution. m^1^A methylation by methyltransferases, including TRMT10C, TRMT6-TRMT61A, and TRMT61B, especially for TRMT61A in this study. And m^1^A on RNA can be removed by ALKBH1 and ALKBH3. As the reader of m^1^A, TDP-43 can recognize and interact with m^1^A, which leads to the mis-localization of TDP-43 to the cytoplasm, further promoting the formation of insoluble TDP-43 inclusion bodies from the liquid phase to the gel-like phase. Conversely, ALKBH3, the demethylase of m^1^A can improve the above effects. (**C**) TDP-43's liquid-to-gel-like transition is facilitated by long CAG repeat RNA expansions.

The next question is how m^1^A promotes aggravated neurodegeneration. TDP-43, the “reader” for m^1^A, was identified as a candidate m^1^A-binding protein by stable isotope labeling by amino acids in the cell culture (SILAC). They found that the RNA-recognition motifs (RRM) domain of TDP-43 binds strongly to m^1^A-containing RNA. TDP-43 was found at a high level in the pull-down mixture of (CAG)_38_, and the TDP-43 level was diminished following ectopic expression of ALKBH3. The expression of TDP-43 is markedly decreased by inhibiting m^1^A in RNA. TDP-43 pathology is characterized by truncation, cytoplasmic mislocalization, and aggregation in the pathological regions of the brain. In the current study, a higher level of truncated endogenous TDP-43 was found in cells expressing (CAG)_38_ RNA or in the striatal tissues of mice expressing Q140 RNA. In addition, endogenous TDP-43 displayed cytoplasmic redistribution upon ectopic expression of (CAG)_22_ and (CAG)_38_, there are larger foci in cells with (CAG)_38_.

Furthermore, the authors found more extensive co-localizations of (CAG)_38_ RNA and TDP-43 than (CAG)_22_ RNA monitored by RNA fluorescence in suit hybridization (FISH) immunofluorescence assays. They found that when ALKBH3 was overexpressed or TRMT61A was genetically depleted, endogenous TDP-43 decreased co-localization with endogenous G3BP1. G3BP1 can also co-localize with (CAG)_38_ RNA by the RNA FISH assay, which may reflect m^1^A in CAG repeat RNA, induce stress granules’ (SGs) formation, and promote the sequestration of TDP-43 into SGs [3] (Fig. 1).

SGs, composed of mRNA and proteins, are membrane-less organelles that dynamically assemble in response to different stress stimuli through LLPS. LLPS is a critical process involving pathological protein aggregation in NDDs, while liquid-to-gel phase separation is an important step between LLPS and protein aggregation. TDP-43 shows abnormal biochemical and biophysical properties in NDDs, and increasing evidence has supported the causal role of TDP-43 assembly via LLPS in neurodegeneration. TDP-43 can generate amyloid filaments, the folds of which characterize distinct neurodegenerative conditions [10]. Impaired TDP-43 LLPS can cause the translocation of excessive cytoplasmic TDP-43 to form SGs [11]. In this study, TDP-43 exhibits phase-separated liquid-like properties in cells, with little recovery of GFP-TDP-43 fluorescence observed after fluorescence after photobleaching (FRAP) in cells with (CAG)_38_ RNA. But ectopic co-expression of ALKBH3 with (CAG)_38_ RNA restored the fast recovery of GFP-TDP-43 fluorescence following photobleaching, suggesting the gel-like property of TDP-43 may be regulated by the interaction of TDP-43 with m^1^A in CAG repeat RNA, in which TDP-43 occurs in phases of separation from liquid-to-gel transitions within SGs. The authors found reduced sensitivity upon treatment with 1,6-hexanediol (1, 6-HD) in cells with (CAG)_38_ RNAs than in cells with (CAG)_22_. These results showed that the longer CAG repeats in RNA can facilitate the TDP-43 phase transition from liquid-to-gel states. At the same length as CAG repeat in RNA, TDP-43 is more susceptible to assembling into gel-like aggregates upon interaction with (CAG)_7_-3m^1^A than incubation with (CAG)_7_ RNA with 0 or 1 m^1^A [3].

Post-transcriptional modifications can mediate SGs’ formation. An intensively studied prototype is the m^6^A-binding YTHDF proteins that promote SGs’ formation [12]. It is striking to note that the current prevalent mechanisms related to RNA methylation are involved in SGs’ formation in NDDs. Given that cellular senescence is the key factor for accelerating the pathology of NDDs, regulating RNA methylation as well as SGs’ formation, indicating that those RNA methylations related to SGs’ formation through LLPS may be viewed as potential pathogenesis of NDDs.

The study by Sun *et al.* revealed a novel pathological role for m^1^A by binding to TDP-43 and exhibiting repeat length-dependent accumulation in CAG repeat RNA. It would be significant to further uncover the commonalities and differences of m^1^A CAG repeat expansions-mediated TDP-43 LLPS in different types of NDDs. m^6^A modification in Chromosome 9 open reading frame 72 (C9orf72) repeat expansions has also been verified to contribute to neurodegeneration [13, 14]. Investigation on the crosstalk among m^1^A, m^6^A, and other RNA modifications could also be conducive to the intervention of NDDs. Besides TDP-43, further studies should also be performed to uncover the role of m^1^A CAG repeat expansions in other important NDDs-associated proteins, such as T cell-restricted intracellular antigen-1 (TIA1) and fused in sarcoma (FUS), which can also undergo LLPS. In CAG repeat expansion disorders, neurotoxicity can be obtained from polyglutamine expansions and in impaired ubiquitin-proteasome systems, suggesting that more pathways for the pathology of NDD initiation induced by nucleotide repeat expansions should be determined in future research. Thus, the current findings shed new light on exploring the pathological mechanisms for a range of NDDs, in addition to CAG repeat expansion-induced neurodegeneration.
